# Vision of the 80s–Reality of the 90s!

**DOI:** 10.1155/S1463924691000081

**Published:** 1991

**Authors:** Francis H. Zenie

**Affiliations:** Zymark Corporation Zymark Center Hopkinton Massachusetts 01748 USA


Journal of Automatic Chemistry, Vol. 13, No. 1 (January–February 1991), pp. 39–42

### Vision of the 80s—Reality of the 90s!

**Francis H. Zenie**
*Zymark Corporation, Zymark Center, Hopkinton, Massachusetts 01748, USA*

### Why this title?

Beginning in the early 1980s, Zymark's founders and pioneering customers shared a vision: they believed that computers and robotics could stimulate a revolution in laboratory operations, leading to dramatic improvements in the quality and timeliness of laboratory results and in staff productivity. Since Zymark was founded, leading organizations throughout the world have installed over 1700 Zymate laboratory Robotics Systems.

Some scientists, however, considered laboratory robotics for their automation needs and concluded that the technology was not yet ready for them. They wanted:

(1) Proven precision and accuracy equal to or better than manual techniques.

(2) Proven reliability in real laboratory operation.

(3) Successful references describing similar applications.

(4) Demonstrated ease of use and rapid start-up.

(5) Assurance of cost-effective results.

(6) Assured access to improved technology and new product developments.

Laboratory robotics is truly an extension of the computer and information management revolution, and, like computer systems, has undergone rapid, evolutionary development. This affinity to the computer industry provides a framework to measure progress and predict trends.

The substantial advancements in laboratory robotics technology during the past decade is making the VISION become REALITY.

### Are we ready for the 90s?

More than ever before, the 1990s will be the decade of increasing challenges, demanded by:

(1) *Global competition.*
This requires: (a) rapid new product development; (b) unsurpassed product quality; and (c) world-class productivity and profitability.

(2) *Government regulation.*
This requires: (a) more analyses; (b) greater sensitivity for trace levels; (c) improved precision; and (d) documented quality assurance.

Successful organizations must meet these challenges. Size or past success does not guarantee future success or even survival. To meet these challenges, laboratories must provide:

(1) *Excellent data for critical business decisions*
Critical decisions which determine product quality, environmental impact, working conditions and process efficiency depend on precise analytical measurements. Superior analytical data are necessary to gain a competitive advantage.

(2) *Unquestioned quality assurance and documentation*
Excellence requires more than good precision. Today's laboratories must 'prove' the validity of analytical results and maintain permanent, secure records.

(3) *Faster analytical results*
Time is our most valuable resource. New products must be introduced faster than those from competing organizations, quality problems must be solved quickly and production lead times minimized. Access to analytical results *cannot* become a bottleneck!

(4) *Improved laboratory productivity*
Today's most productive laboratories re-allocate work between people, automated instruments, workstations and computers to gain maximum effectiveness from their most valuable resource—skilled people.

(5) *More effective management and staff*
Every day, first line laboratory managers are challenged to *provide quality analytical data, on time, and with existing staff.* The need for qualified laboratory staff is increasing, while fewer people are being trained in chemistry and related sciences. Innovative allocation of tasks between people and automated instrumentation not only improves results but helps attract and motivate good people.

Zymark are often asked to accept the following advice: 'If it ain't broke, don't fix it'. This should not be accepted. Leading managements are pro-active. They anticipate potential problems and convert them into opportunities.

*Fortune* magazine, in the 26 March, 1990 issue, collected important ideas for the 1990s in an article called 'Today's leaders look to tomorrow'. Comments by recognized business leaders highlight critical concerns for everyone. John F. Welch, Jr, Chief Executive of General Electric, defined the issues and urgency.

> The pace of change in the 90s will make the 80s look like a picnic—a walk in the park. Competition will be relentless. The bar of excellence in everything we do will be raised every day . . . Technological innovation and the translation of that innovation into marketplace advantage will be accelerating ever faster. And in the coming decade, we're going to see increasing demands for sensitivity to the environment.

> Simply doing more of what worked in the 80s . . . will be too incremental. More than that, it will be too slow. The winners of the 90s will be those who can develop a culture that allows them to move faster, communicate more clearly, and involve everyone in focused effort to serve evermore demanding customers.

### Technology: a tool to meet the challenges

Zymark is proud of its pioneering developments in laboratory automation and continuing improvements and additions to this technology. However, *Technology alone is not the solution. Technology is a tool used by people to solve problems and make things better.*

The current short-term approach needs to be replaced with a strategic commitment to automation. Tom Peters in *Thriving on Chaos*, highlighted one area of this dilemma:

> We are misusing automation. Americans still see it as a tool to reduce the need for labor, not as a tool to aid labor in adding value to the product.

Rather than define laboratory automation in terms of technology, the criteria for successful laboratory automation should be defined. Successful laboratory automation: (a) assures precise and timely analytical results; (b) uses valuable people's time more effectively; and (c) quickly recovers the automation investment through competitive advantage and improved productivity.

Laboratory automation began with the early developments in instrumental analysis. The chemical, pharmaceutical and related industries could not exist in today's form without ongoing innovations in analytical chemistry. Computers and low-cost microprocessors became the engine for even greater advances.

Laboratory robotics, pioneered by Zymark's Zymate System in 1982, opened the door to automation of sample preparation and other laboratory procedures. The goal was to provide flexible, modular technology that could be tailored by end-users to meet their specific needs.

### Laboratory robotics

As with successful computer systems, laboratory robotics requires a powerful, underlying architecture to attain optimum performance and assure compatibility to new developments.

*Zymate's Architecture* is a platform that supports ongoing development of this powerful automation technology. The pioneering role of this new technology created a need for continuous improvement, building on experience gained performing applications under actual laboratory conditions. The architecture enables *Zymate Systems to meet today's requirements and offer the power and flexibility to address tomorrow's needs.*

Zymate architecture creates:

(1) *A powerful, lasting foundation.* The Zymate System's unique hardware and software architecture assures:

(a) *Modularity*: hardware and software modules that automate specific laboratory operations are guaranteed to work together in an integrated system.

(b) *Flexibility*: systems can be reconfigured or expanded to meet the changing needs of the laboratory.

(c) *Meeting your needs*: the structure is well defined and documented, and yet open for easy access to powerful system features. Users may modify capabilities to meet their specific needs. Zymate Systems can interface directly to a wide range of automated instruments from multiple vendors.

(2) *Access to state-of-the-art technology.* The open architecture and modular design allow new technical advances to be adopted and implemented quickly in both new and installed Zymate Systems. This enables Zymark to provide access to ongoing developments.

(3) *A platform for the future.* Most of the standard and custom Zymate modules were either not available or have undergone significant development since the Zymate System was first introduced. Through this evolution, the Zymate architecture remained intact and continues to guarantee a powerful foundation for further development.

### *State-of-the-art now!*

Several recent developments illustrate the power of today's state-of-the-art technology and its unique platform for even further enhancements.

*PyTechnology*—extends the Zymate architecture to create an advanced, high-level application environment. The standardized hardware and software incorporates proven, reliable laboratory techniques to ensure high-quality results, rapid installation and ease of use.

*System V Controller*—links laboratory robotics and the information management revolution. Implements all user interfaces, program and data files in industry standard, IBM PC compatible formats. Permits full integration with networks and information management installations.

*Gravimetric Laboratory Techniques (GLTs)*—assure high-quality results through complete documentation and automatic audit trail. GLTs use precision electronic balances to measure, control and report liquid transfer steps in laboratory methods.

GLTs gained wide acceptance as a powerful alternative to human eyesight in manual procedures. Using GLTs, automated systems will:

(a) Perform all liquid handling steps to new standards of precision.

(b) Detect and alert to the presence of potential outliers from such sources as empty reagent reservoirs, leaking syringes, loose fittings, plugged lines or air bubbles.

(c) Assure high-quality results by reporting a complete audit trail, including: positive sample ID from barcode or keyboard entry, sample weight and all actual liquid handling, mass-transfer steps.

*AccuTrak*—Zymark's patented technology, AccuTrak, enables Zymate robots to maintain outstanding precision during months and years of use. All robotics technology requires accurate position feedback capability, called servomechanisms, to ensure precise, reliable positioning. For years, engineers have debated the respective advantages and disadvantages of digital encoders and analogue potentiometers as feedback techniques. AccuTrak *combines* analogue potentiometer, digital encoder and microprocessor technology to form a self-calibrating feedback device for reliable, accurate position control. Only AccuTrak offers the continuous resolution and absolute position reference of potentiometers and the excellent linearity and minimum wear from digital encoders.

These innovations bring laboratory robotics to new levels of precision, reliability and ease of use compared with early use of the technology.

### Multiple automation solutions

The wide range of laboratory needs demand multiple approaches to laboratory automation: integrated robotic systems; and laboratory workstations—individual or multiple. Laboratory needs will dictate the most effective approach and laboratories may use several approaches to meet different needs. The following definitions help explain the alternatives:

*Integrated robotic systems* automate complete procedures, often including sample introduction into the analytical instrument. Zymark's robotic systems use a modular approach to configure Laboratory Unit Operations into a complete system tailored to specific laboratorys' needs.

*Laboratory workstations* are personal automation tools that assist people in performing their job better and more efficiently. Zymark's workstations provide well defined, standardized combinations of sample preparation functions, which perform portions of procedures common to many methods.

*Integrated robotic systems*

Integrated robotics systems are well suited to centralized laboratories with large sample loads. Integrated systems are most effective when unattended automation of entire procedures are desired for:

(1) Complex, technique dependent procedures.

(2) Varying sample loads requiring standby capacity for peak demands.

(3) Around-the-clock operation to ensure timely results.

(4) Unstable samples that must be analysed immediately following sample preparation.

(5) Assured 'chain of custody' by eliminating human intervention.

Analytical instruments are integrated into robotic systems when: (a) the measurement is fast relative to sample preparation; (b) the analytical instrument is relatively inexpensive; and (c) system use is high—6 or more hours per day.

*Laboratory workstations: personal automation for sample preparation and autosampling*

Laboratory workstations offer a cost-effective approach to automating common steps in laboratory procedures. They are the next step beyond chromatographic autosamplers and an excellent path to higher automation levels. Workstations free people from routine tasks and permit them to perform data analysis and other value-added work.

Using well defined, standardized techniques, laboratory workstations deliver outstanding precision and reliability in addition to low cost and ease of use. Laboratory workstations are most effective in decentralized laboratories which analyse a wide range of samples using many methods of sample preparation and analysis.

Multiple laboratory workstations create an even more flexible alternative to laboratory automation. Each workstation performs its intended function and people manage the flow of work between workstations. This distribution of tasks leads to very cost-effective investment and excellent utilization of valuable people. Figures 1–3 show the use of multiple workstations for pharmaceutical dosage form analysis.

Figure 1 illustrates a typical flow of work between two workstations. In this example, the Tablet Processing Workstation prepares tablet or capsule samples, ready for final clarification, dilution and analysis. The BenchMate Workstation performs ultraprecise dilutions and filtration for HPLC or UV/Vis analysis. Laboratory flexibility is enhanced because the BenchMate and HPLC are easily accessible for other work when available.

Figure 2 shows multiple workstations using alternate work flow paths to match HPLC conditions, analysis timing and HPLC availability.

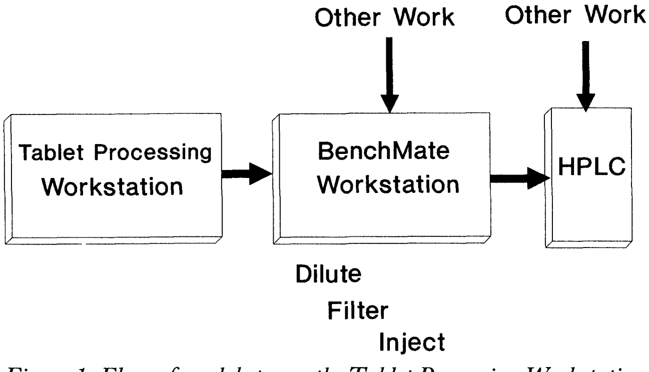

*Figure 1. Flow of work between the Tablet Processing Workstation and the BenchMate Workstation.*

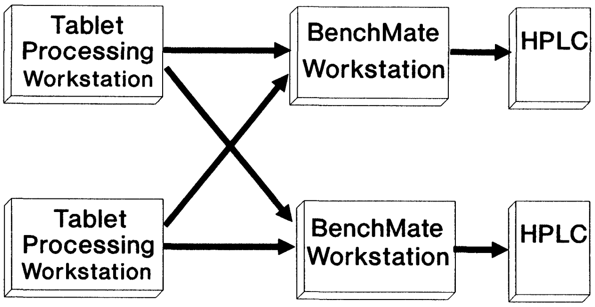

*Figure 2. Flow of work between multiple workstations.*

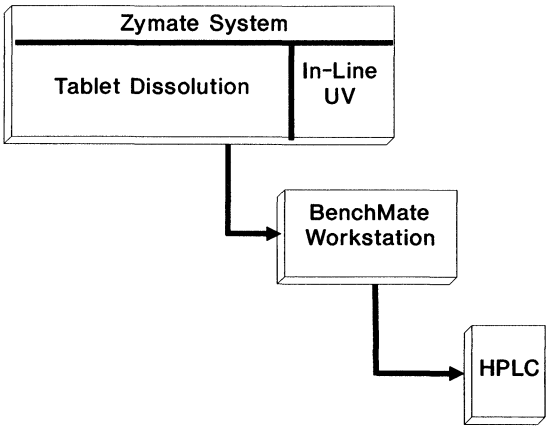

*Figure 3. Integrated robotics system for dissolution testing.*

Figure 3 shows an integrated robotics system for dissolution testing for both UV and HPLC analysis. The alternate path to the BenchMate Workstation permits off-line HPLC analysis, in addition to in-line UV analysis.

### Technology transfer

Laboratory robotics and laboratory workstations complement each other to provide valuable alternatives to meet laboratory automation needs. Experience gained from successful Zymate installations helps define critical applications and common needs. The flexible, modular Zymate architecture makes it an excellent development platform for future workstations.

Laboratory workstations provide the standard product opportunity to invest engineering resources for superior performance and reliability. The improved, standardized workstation techniques can then be migrated back into the flexible robotics realm.

### Technology trends

The needs for improved technology are clear and define several key trends, including:

- High reliability.
- Improved ease of use.
- Higher throughput:
  - active modules—less dependent on the robot;
  - faster program processing;
  - faster robotics.
- More powerful modules.
- New laboratory workstations.
- Improved integration with laboratory computers and instruments.

Laboratory robotics is still a young, emerging technology. Many pioneers have made their contribution and more breakthroughs will come. Today's products perform better, are easier to use and are more reliable than ever before—and they will get better.

### Beyond technology: partners for success

Zymark believe that the greatest benefits from powerful laboratory automation technology will be realized through strategic alliances between quality vendors and customers. Successful implementation of laboratory automation cannot be achieved as a simple sales transaction, but through a long-term relationship built on trust, aligned goals and mutual payback.

Even before a sale is made, the partnership defines special roles, including:

(1) The customer must teach and Zymark must understand the customer's needs—technical, business and people related.

(2) Zymark must educate the customer about alternative solutions and their advantages and disadvantages.

(3) Zymark must recommend approaches that offer the highest value, and help the customer make an informed decision. Based on good advice, the customer must decide if the available solutions offer more value than the investment.

Advanced technology plus new and stronger partnerships will lead to new standards of laboratory performance and productivity. Zymark are proud to be helping our customers meet their challenges of the 1990s!

### Acknowledgement

This paper was presented at the International Symposium on Laboratory Automation and Robotics (Boston, USA, 16–19 September 1990).

